# Significant Association of *Streptococcus bovis* with Malignant Gastrointestinal Diseases

**DOI:** 10.1155/2011/792019

**Published:** 2011-10-29

**Authors:** Salah Shanan, Samia A. Gumaa, Gunnar Sandström, Hadi Abd

**Affiliations:** ^1^Division of Clinical Microbiology, Department of Laboratory Medicine, Karolinska Institute, Karolinska University Hospital, Huddinge, 141 86 Stockholm, Sweden; ^2^Faculty of Medical Laboratory Sciences, University of Medical Sciences and Technology, Khartoum 11111, Sudan; ^3^Department of Microbiology and Parasitology, Faculty of Medicine, University of Khartoum, Khartoum 11111, Sudan

## Abstract

*Streptococcus bovis* is a Gram-positive bacterium causing serious human infections, including endocarditis and bacteremia, and is usually associated with underlying disease. The aims of the current study were to compare prevalence of the bacterium associated with malignant and nonmalignant gastrointestinal diseases and to determine the susceptibility of the isolated strains to different antimicrobial agents. The result showed that the prevalence of *S. bovis* in stool specimens from patients with malignant or with nonmalignant gastrointestinal diseases was statistically significant. This result may support the idea that there is correlation between *S. bovis* and the malignant gastrointestinal diseases.

## 1. Introduction

Several species of bacteria have been linked to chronic infections of colon and have been shown to increase the risk of colon cancer. *Streptococcus bovis *has been evaluated as one of the possible etiologic agents for colorectal cancer [1].

*S. bovis *is a Gram-positive bacterium, which is considered as a normal inhabitant of the human gastrointestinal tract but is less frequently present than other *Streptococcus* species [2]. Whatever, *S. bovis* has been shown to be an increased cause of endocarditis and bacteraemia and found to be associated with gastrointestinal diseases [3–6] or with the colorectal cancer [7–10].

Although a number of bacteria have been associated with cancer, their possible role in carcinogenesis is unclear. In some cases *Helicobacter pylori* may cause stomach cancer [11, 12] since animal models have demonstrated Koch's third and fourth postulates for the role of *H. pylori* in the causation of stomach cancer [13]. Moreover, it has been found also that *Salmonella typhi* is associated with gallbladder cancer [14] and *Escherichia coli with *Crohn's disease as well as colon cancer [15, 16]. 

McCoy and Mason first reported a case of the association between streptococcal endocarditis and colon cancer in 1951 [7]. Many studies have examined the presence of *S. bovis* in stool samples obtained from patients with colorectal cancer to find a relationship between this bacterium and the risk of colorectal cancer. In 1977, Klein et al. found a significantly strong association of *S. bovis *in stool samples of patients with colon cancer compared with healthy controls and patients with nonmalignant gastrointestinal disease [17]. This important finding was confirmed in later studies [18–20]. In contrast, other studies did not find any significant association between faecal *S. bovis* and human colorectal cancer [21–24].

The aims of the current study were to isolate *S. bovis* from stool specimens collected from patients with malignant and non-malignant gastrointestinal diseases and from a healthy group to compare the association of the bacterium in malignant and non-malignant gastrointestinal diseases and to determine the susceptibility of the isolated strains to different antimicrobial agents. 

## 2. Materials and Methods

### 2.1. Study Area and Population

Stool specimens were collected in sterile containers before the initiation of therapy from inpatients with malignant and non-malignant gastrointestinal diseases in Ibn-Sina Specialized Hospital, Khartoum, Sudan. The stool samples were from 28 inpatients with malignant gastrointestinal diseases (Table 1), 27 inpatients with non-malignant gastrointestinal diseases (Table 2), and 50 controls. All the patients and controls were men.

20 out of the 50 controls were outpatients who came to the gastric intestinal tract clinic suffering from the gastrointestinal tract problems and the medical examination and laboratory tests showed that they did not have any type of cancer.

The inpatients and the 20 controls were working in agriculture and animal husbandry and aged between 25 and 65 years. 5% of each inpatients and controls aged from 25 to 50 years and 95% aged from 51 to 65 years.

30 out of the 50 controls were apparently-healthy farmers aged 50–86 years and the mean age was 64 ± 10 years. They had no history of taking any antimicrobial agent for at least one month. The stool samples were collected during the medical survey for infectious and tropical diseases organized by University of Medical Sciences and Technology in Sudan from Gezira agriculture Scheme with a population of 40 thousand people.

### 2.2. Culture of Stool Sample and Identification of Growing Bacteria

The stool samples were cultured on MacConkey agar plates (Himedia Limited, Bombay) and incubated aerobically at 37°C overnight. Identification of growing bacteria was performed according to standard bacteriological methods.

### 2.3. Susceptibility of Identified Bacteria to Antibiotics

Antimicrobial susceptibility to penicillin and trimethoprim-sulfamethoxazole was carried out on the isolated strains by disk diffusion method on Mueller-Hinton agar plates (Himedia Limited, Bombay). Sensitivity of bacteria to each antibiotic was carried out by measuring the diameter of inhibition of bacterial growth around the disc. A diameter of ≥26 mm was considered as the cut-off point for sensitivity for the penicillin and a diameter of ≥24 mm for the trimethoprim-sulfamethoxazole according to National Committee for Clinical Laboratory Standard (NCCLS) method.

### 2.4. Statistical Analysis

*χ* ^2^ test was used for significant prevalence of *S. bovis* in stool specimens from patients with malignant or with non-malignant gastrointestinal diseases.

The statistical analysis was also performed to compare between presence of *S. bovis* in stool specimens from patients with noncolonic cancer and with non-malignant gastrointestinal diseases.

## 3. Results

### 3.1. Susceptibility of *S. bovis* Strains to Antibiotics

Stool specimens were collected from fifty-five patients with malignant and non-malignant gastrointestinal diseases and from fifty persons as control group. The specimens were examined to the prevalence of *Streptococcus bovis* strains and their susceptibility to the used antibiotics.

All the isolated *S. bovis* strains were found to be sensitive to penicillin and trimethoprim-sulfamethoxazole with diameter of inhibition from 26 to 32 mm and from 24 to 32 mm, respectively. *S. bovis* was not detected in the control group but it was detected in the inpatients aged 51–65 years.

### 3.2. Prevalence of *S. bovis* in Malignant Gastrointestinal Diseases

Bacteriological analysis of stool specimens from the patients with malignant gastrointestinal diseases showed that out of 28 specimens *S. bovis* was isolated from 10 patients. Six positive were from patients with carcinoma of the stomach, and one positive was from each patient with carcinoma of rectum, pancreas, colon, and liver. The prevalence of *S. bovis *in stool from the patients with carcinoma in stomach, rectum, pancreas, colon, polyps, and hepatocytes was 55%, 25%, 14%, 50%, 0%, and 50%, respectively (Table 3).

### 3.3. Prevalence of *S. bovis* in Nonmalignant Gastrointestinal Diseases

Analysis of stool specimens from the patients with non-malignant gastrointestinal diseases showed that out of 27 specimens *S. bovis* was isolated from 5 patients. Two positive from patients with liver cirrhosis, two positive from patients with obstructive jaundice, and one from patient with portal hypertension were found. The prevalence of *S. bovis *in stool from the patients with Crohn's disease, liver cirrhosis, obstructive jaundice, portal hypertension, and viral hepatitis was 0%, 28%, 33%, 14%, and 0%, respectively (Table 4).

### 3.4. Prevalence of *S. bovis* in Stool from the Patients with Malignant and Nonmalignant Gastrointestinal Diseases and Healthy Controls

**Figure 1:**
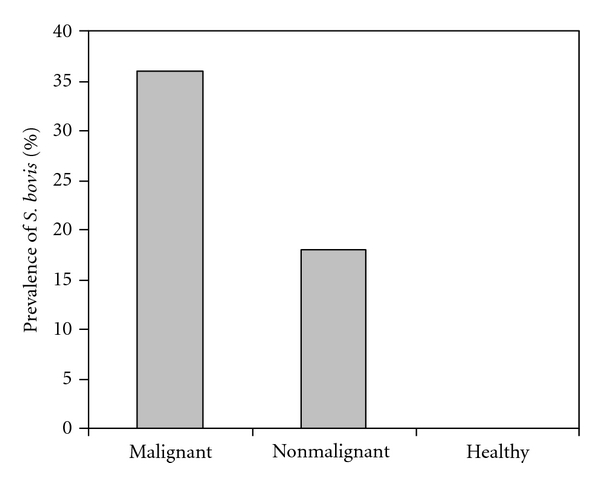
Prevalence of *S. bovis* in stool from the patients with malignant and non-malignant gastrointestinal diseases and healthy controls.

From analysing the prevalence of *S. bovis* in stool from the patients with malignant and non-malignant gastrointestinal diseases and healthy controls was 36%, 18%, and 0%, respectively (Figure 1). The statistical analysis showed that the prevalence of *S. bovis* in stool specimens from patients with malignant or with non-malignant gastrointestinal diseases was statistically significant (*P* value of *χ* ^2^ test was 0.02).

## 4. Discussion

Colorectal cancer is the fourth most common cancer among men and third most common among women worldwide [25]. It was reported that infectious agents accounted for 18% of all cancers worldwide [26].* S. bovis* is Gram-positive bacterium causing serious human infections. A large number of previous studies point to association of *S. bovis* with gastrointestinal diseases [5, 6] and cancer of the human colon [10].

The current study finds a correlative relationship between existence of *S. bovis* and malignant gastrointestinal diseases since prevalence of the bacterium in stool from the patients with carcinoma in stomach, rectum, colon, and hepatocytes was 55%, 25%, 50%, and 50%, respectively. In comparison, the prevalence of *S. bovis *in stool from the patients with Crohn's disease, liver cirrhosis, obstructive jaundice, portal hypertension, and viral hepatitis was 0%, 28%, 33%, 14%, and 0%, respectively. 

From analysis the prevalence of *S. bovis* in stool from the patients with malignant and non-malignant gastrointestinal diseases and healthy controls was 36%, 18%, and 0%, respectively. The *χ* ^2^ test statistical analysis showed that the prevalence of *S. bovis* in stool specimens from patients with malignant or with non-malignant gastrointestinal diseases was statistically significant (*P* = 0.02). The current findings confirm the previous data that correlated the association of *S. bovis* with colorectal cancer specially Klein study 1977 [17] and the later studies [18–20].

In contrast to the literature regarding an association with colorectal cancer, less is written about associations of *S. bovis *with other gastrointestinal diseases or with other cancers except Klein study 1977 [17] but Klein 1987 reported also the lack of association of *S. bovis* with noncolonic gastrointestinal carcinoma [27]. However, Alazmi et al., 2006 found that *S. bovis* bacteremia in adults was frequently associated with hepatic dysfunction [28], and Zarkin et al., postulated a triad of *S. bovis *bacteremia, liver disease, and colonic pathology whereby the liver disease might account for the increased fecal carriage, entry to the portal venous system, or passage from the portal to systemic circulation of *S. bovis* [29].

In this context, our findings add more information about the association of *S. bovis* with non-malignant gastrointestinal diseases and with non-colonic cancer: since the current study isolated *S. bovis *from the stool of patients with liver cirrhosis, obstructive jaundice, and portal hypertension (Table 4) and the bacterium was isolated from stool of patients with carcinoma in stomach, rectum, and hepatocytes (Table 3), the association of *S. bovis* with non-malignant gastrointestinal diseases or with noncolonic cancer was not significantly different by *χ* ^2^ test (*P* > 0.05). However, Klein 1977 isolated *S. bovis* from stool of patients with inflammatory bowel disease, other gastrointestinal disorders and noncolonic neoplasms [17] and he found that presence of *S. bovis* in stool of patients with non-colonic cancer was not significantly different from that in controls [27].

Tjalsma and colleagues detected immune reactions against *S. bovis* antigens in sera of 11 out of 12 colon cancer patients and in 3 out of 4 patients with colon polyps. No positive reaction was observed in 8 control subjects [30]. They found that one of the diagnostic antigens represents a surface-exposed heparin-binding protein that, according to their speculation, might be involved in the attachment of *S. bovis* to tumor cells. They claim that profiling of the humoral immune response against *S. bovis* infections may represent a promising diagnostic tool in early detection of human colon cancer. However, research has not yet determined if *S. bovis* is a causative agent of colon cancer or if preexisting cancer makes the lumen of the large intestine more hospitable to *S. bovis* outgrowth [31].

Previous findings (reviewed in [31]) suggest an active role of *S. bovis* in the promotion of intestinal carcinogenesis when adult rats were treated with azoxymethane for 2 weeks and subsequently received injections with either* S. bovis* bacteria or wall-extracted antigens twice weekly. The authors observed progression of preneoplastic lesions, enhanced expression of proliferation markers, and increased production of interleukin-8 in the colonic mucosa in these rats [32]. The same group used a partially purified *S. bovis* S300 fraction representing 12 different proteins and triggered the synthesis of proinflammatory proteins (human interleukin-8 and prostaglandin E2), correlated with the *in vitro* overexpression of cyclooxygenase-2 in human colon carcinoma cells and in rat colonic mucosa [33]. These data could point to a role of oxygen radicals in colon carcinogenesis induced by a chronic infection with *S. bovis*. The mechanism could be similar to the one suspected for the development of gastric carcinomas after persisting *H. pylori* infections since a cecropin-like *H. pylori* peptide, Hp (2–20), was found to be a monocyte chemoattractant and activated the monocyte NADPH oxidase to produce oxygen radicals [34].

Presently it would still be important to know whether the increased presence of *S. bovis* in colonic cancers and polyps results from the preferential bacterial colonization of these cancers and their precursors or whether *S. bovis* represents a carcinogen that is causally involved in gastrointestinal cancer [31]. However, our study presents a significant association of *Streptococcus bovis* with malignant gastrointestinal diseases.

## 5. Conclusions

The significant association of *S. bovis* with malignant gastrointestinal diseases compared to its association with non-malignant gastrointestinal diseases presented in this study confirms the previous studies about the association between this bacterium and colorectal cancer and may support the idea that there is correlation between this bacterium and the malignant gastrointestinal diseases.

## Figures and Tables

**Table 1:** Stool specimens from malignant gastrointestinal diseases.

Malignant diseases	Number of examined stool specimens
Carcinoma of the stomach	11
Carcinoma of the rectum	4
Carcinoma of the pancreas	7
Carcinoma of the colon	2
Hepatocellular carcinoma	2
Premalignant Polyp	2

Total	28

**Table 2:** Stool specimens from non-malignant gastrointestinal diseases.

Non-malignant diseases	Number of examined stool specimens
Crohn's disease	4
Liver cirrhosis	7
Obstructive jaundice	6
Portal hypertension	7
Viral hepatitis	3

Total	27

**Table 3:** Prevalence of *S. bovis* isolated from patients with malignant gastrointestinal diseases.

Malignant Diseases	Number of examined stool specimens	Number of positive stool specimens	Prevalence of *S. bovis *%
Carcinoma of the stomach	11	6	55
Carcinoma of the rectum	4	1	25
Carcinoma of the pancreas	7	1	14
Carcinoma of the colon	2	1	50
Pre-malignant Polyp	2	0	0
Hepatocellular carcinoma	2	1	50

Total	28	10	36

**Table 4:** Prevalence of *S. bovis* isolated from patients with non-malignant gastrointestinal diseases.

Non-malignant diseases	Number of examined stool specimens	Number of positive stool specimens	Prevalence of *S. bovis *%
Crohn's disease	4	0	0
Liver cirrhosis	7	2	28
Obstructive jaundice	6	2	33
Portal hypertension	7	1	14
Viral hepatitis	3	0	0

Total	27	5	18
